# Association Between Clinical Dysphagia Assessment Tools and Videofluoroscopic Findings in Amyotrophic Lateral Sclerosis: A Retrospective Study

**DOI:** 10.3390/medicina62061039

**Published:** 2026-05-27

**Authors:** Burak Manay, Demet Aygün, Alperen Şentürk, Mustafa İbas, Ramazan Güven, Şeyda Belli

**Affiliations:** 1 Department of Speech and Language Therapy, Faculty of Health Sciences, Istanbul Atlas University, Istanbul 34403, Turkey; alperen.senturk@atlas.edu.tr; 2 Department of Neurology, Faculty of Medicine, Istanbul Atlas University, Istanbul 34403, Turkey; demet.aygun@atlas.edu.tr; 3 Department of Otolaryngology—Head and Neck Surgery, Istanbul Atlas University, Istanbul 34403, Turkey; mustafa.ibas@atlas.edu.tr; 4 Department of Emergency Medicine, Faculty of Medicine, Istanbul Atlas University, Istanbul 34403, Turkey; ramazan.guven@atlas.edu.tr; 5 Department of Otolaryngology, Bağcılar Training and Research Hospital, University of Health Sciences, Istanbul 34200, Turkey; seyda.belli@sbu.edu.tr

**Keywords:** amyotrophic lateral sclerosis, clinical swallowing assessment, dysphagia, Penetration–Aspiration Scale, videofluoroscopic swallowing study

## Abstract

*Background and Objectives*: Amyotrophic lateral sclerosis (ALS) is a neurodegenerative disease frequently associated with dysphagia and aspiration risk. This study aimed to investigate the relationship between clinical dysphagia assessment tools (EAT-10, GUSS, RSST, and sialorrhea severity) and videofluoroscopic swallowing study (VFSS) findings in patients with ALS. *Materials and Methods*: This retrospective observational study included 60 patients with ALS classified as spinal-onset (*n =* 38) or bulbar-onset (*n =* 22). Relationships between clinical assessments and VFSS findings were analysed using Spearman correlation analysis. Exploratory multivariable regression and receiver operating characteristic (ROC) analyses were performed to evaluate associations and aspiration risk discrimination. *Results*: Strong negative correlations were observed between PAS–Liquid and RSST and GUSS scores, whereas EAT-10 showed a strong positive correlation (all *p* < 0.001). ROC analyses demonstrated good discriminative ability for aspiration risk for GUSS (AUC = 0.89), RSST (AUC = 0.88), and EAT-10 (AUC = 0.82). Patients with bulbar-onset ALS demonstrated higher penetration–aspiration severity and lower functional oral intake. *Conclusions*: Clinical dysphagia assessment tools showed significant associations with instrumental swallowing findings in ALS. GUSS and RSST demonstrated good discriminative ability for aspiration risk and may be clinically useful bedside screening tools. However, instrumental swallowing assessment remains essential whenever feasible.

## 1. Introduction

Amyotrophic lateral sclerosis (ALS) is a neurodegenerative disorder characterized by the progressive degeneration of upper and lower motor neurons and a high mortality rate [1,2]. Bulbar muscle involvement is common, and dysarthria, dysphagia, and sialorrhea frequently accompany the clinical presentation [3]. Dysphagia develops in the majority of patients and negatively impacts survival by increasing the risk of malnutrition and aspiration pneumonia [4,5,6]. Impaired bolus control due to oral and pharyngeal muscle weakness, along with inadequate laryngeal closure, may lead to penetration and aspiration [7,8,9]. The pathophysiology of dysphagia involves both upper and lower motor neuron degeneration [10,11].

Approximately 60–70% of ALS cases have a spinal onset, whereas 25–30% present with a bulbar onset [12]. Dysphagia tends to occur earlier in patients with bulbar-onset ALS, while it generally develops later in those with spinal-onset disease [13]. With disease progression, the risk of aspiration increases, and respiratory muscle involvement significantly increases mortality [14]. These variations in clinical progression suggest that onset type may affect the performance of clinical dysphagia assessment tools.

In ALS, involvement of the bulbar muscles may lead to impairments in oral motor function. Oral motor dysfunction is considered one of the early indicators of dysphagia. Schimmel et al. (2021) [15] reported reduced chewing performance and decreased lip and tongue strength, along with increased Eating Assessment Tool-10 (EAT-10) scores, in patients with ALS. Despite a reduction in spontaneous saliva production, difficulties in saliva control may still be observed, suggesting that sialorrhea may be more closely related to decreased swallowing frequency rather than increased secretion [15].

These findings suggest that dysphagia may emerge early in ALS and underscore the importance of clinically evaluating swallowing function. In this context, subjective scales and clinical tests are commonly used in the assessment of dysphagia in ALS [6,16]. The videofluoroscopic swallowing study (VFSS) is the gold standard for assessing swallowing safety, and the Penetration–Aspiration Scale (PAS) and the Functional Oral Intake Scale (FOIS) provide objective measures [17].

In recent years, several bedside dysphagia assessment tools have been investigated in both ALS and other neurological populations. The Eating Assessment Tool-10 (EAT-10) is widely used for evaluating patient-reported dysphagia symptoms, whereas the Gugging Swallowing Screen (GUSS) has demonstrated good sensitivity for aspiration screening, particularly in stroke populations. Similarly, the Repetitive Saliva Swallowing Test (RSST) has been associated with swallowing safety and aspiration risk in neurological disorders. In addition, sialorrhea is a common clinical finding in ALS and may reflect impaired oral motor control and reduced swallowing frequency. Furthermore, the Functional Oral Intake Scale (FOIS) is frequently used to evaluate functional oral intake level and feeding dependence in dysphagic patients. However, despite the widespread clinical use of these bedside assessment tools, studies directly comparing multiple clinical dysphagia assessment tools with instrumental VFSS findings in patients with ALS remain limited. In particular, the discriminative ability of these tools for identifying aspiration risk in ALS has not been sufficiently investigated.

Although the EAT-10, the Gugging Swallowing Screen (GUSS), and the Repetitive Saliva Swallowing Test (RSST) are widely used in clinical practice, their agreement with VFSS findings and their variation according to onset type remain unclear [6,16]. This study aimed to investigate the relationship between clinical dysphagia assessment tools and VFSS findings in patients with ALS, and to identify the assessment tool that most accurately reflects swallowing safety and functional oral intake in clinical practice.

## 2. Materials and Methods

### 2.1. Study Design and Setting

This single-centre retrospective observational study reviewed medical records of patients with ALS followed between November 2022 and December 2025 at Istanbul Atlas University Hospital. The hospital is a multidisciplinary centre where neurology, otolaryngology, and speech and language therapy departments collaborate in the evaluation and management of dysphagia in neurological diseases. Clinical dysphagia assessments (EAT-10, GUSS, RSST, and sialorrhea) and videofluoroscopic swallowing study (VFSS) findings obtained during routine care were analysed retrospectively. The study was approved by the Atlas University Non-Interventional Ethics Committee (Approval No: 01/15; Date: 26 January 2026; Ref No: E-22686390-050.99-90309) and conducted in accordance with the Declaration of Helsinki.

### 2.2. Study Population

**Figure 1:**
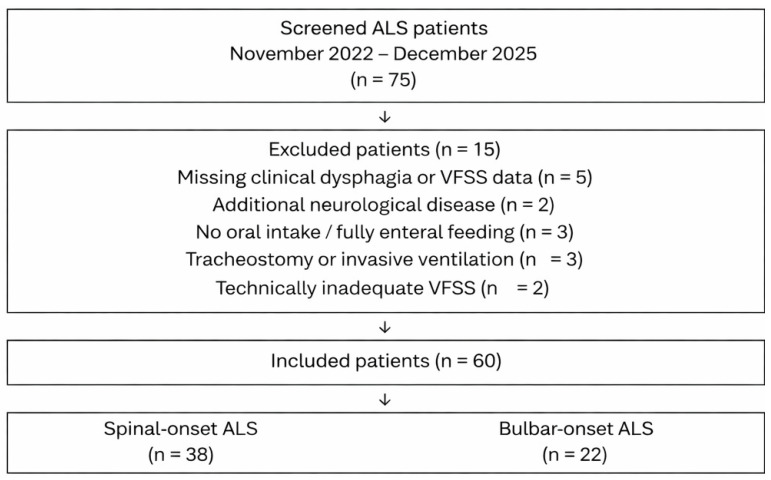
Flowchart of ALS patients included in the study.

The study sample was determined by reviewing the medical records of patients who met the inclusion criteria during the specified time period. A total of 60 patients with ALS were included. The patient selection process is illustrated in Figure 1. Participants were classified as spinal-onset (*n* = 38) or bulbar-onset (*n* = 22).

Inclusion criteria were: age ≥ 18 years; a confirmed diagnosis of ALS according to the revised El Escorial criteria; follow-up at Istanbul Atlas University Hospital between November 2022 and December 2025; availability of at least one clinical dysphagia assessment (EAT-10, GUSS, RSST, or sialorrhea assessment); and completion of VFSS with recorded PAS and FOIS scores. Patients with partial oral intake, including those supported with percutaneous endoscopic gastrostomy (PEG), were included.

Exclusion criteria were: age < 18 years; juvenile ALS; missing clinical or VFSS data; additional neurological or structural conditions affecting swallowing; complete dependence on enteral feeding without oral intake; presence of tracheostomy or invasive mechanical ventilation; and non-interpretable VFSS recordings.

### 2.3. Data Sources

Patients were identified through the Hospital Information Management System using ICD-10 codes for ALS (G12.21) and dysphagia (R13.1). Demographic and clinical data, including age, sex, ALS onset type, disease duration, ALSFRS total score, and nutritional status (oral, PEG-supported, or NG tube), were extracted from medical records. Clinical dysphagia assessments and VFSS examinations were performed on the same day as part of routine multidisciplinary dysphagia evaluation. VFSS recordings were independently reviewed by two experienced speech and language therapists who were blinded to the clinical assessment scores, and Penetration–Aspiration Scale (PAS) scores were assigned. Inter-rater reliability was assessed using the intraclass correlation coefficient (ICC) with a two-way random-effects model and absolute agreement.

### 2.4. Instrumental Assessment of Swallowing Function

VFSS recordings obtained during routine follow-up were analysed for objective assessment of swallowing function. Evaluations were performed using a General Electric Precision RXi fluoroscopy system (GE Healthcare, Chicago, IL, USA), with patients positioned upright in lateral view and recordings acquired at 30 frames per second.

Standard protocol included liquid and semi-solid consistencies. Liquid boluses were administered as 3 × 5 mL with volume adjustments based on safety, while semi-solids were given in 5 mL portions, along with a single bite of solid bread.

For each consistency, the highest PAS score was recorded as the representative value. The Penetration–Aspiration Scale (PAS), developed by Rosenbek et al. (1996), is an 8-point scale assessing airway invasion, with higher scores indicating poorer airway protection [18]. The validity and reliability of the scale in Turkish were established by Karaduman et al. (2012) [19]. In the analyses, the variables “PAS-Liquid” and “PAS-Semi-solid” were used for liquid and semi-solid consistencies, respectively.

FOIS is a valid and reliable scale that classifies an individual’s level of oral intake from 1 (nothing by mouth) to 7 (full oral intake) [20]. FOIS scores were obtained from patient records and used in the analyses.

### 2.5. Clinical Dysphagia Assessment Tools

#### 2.5.1. Gugging Swallowing Screen (Guss)

GUSS is a bedside dysphagia screening tool used to assess aspiration risk, consisting of indirect and direct swallowing assessments. It is scored between 0 and 20, with higher scores indicating better swallowing performance. The Turkish version of the scale has been shown to be valid and reliable [21].

#### 2.5.2. Eating Assessment Tool-10 (Eat-10)

EAT-10 is a 10-item self-reported questionnaire used to subjectively assess dysphagia symptoms. The total score ranges from 0 to 40, with scores ≥ 3 considered indicative of dysphagia risk. The Turkish version of the scale has been shown to be valid and reliable [22].

#### 2.5.3. Repetitive Saliva Swallowing Test (Rsst)

RSST is a clinical test used to assess swallowing control and endurance. The individual is instructed to swallow their own saliva as many times as possible within 30 seconds, and the number of swallows is recorded. Fewer than three swallows within 30 seconds suggests the need for a more detailed evaluation of swallowing function [23]. In this study, existing clinical records were analysed.

### 2.6. Sialorrhea Assessment

Sialorrhea data were retrospectively obtained from clinical examination and follow-up records. Severity was evaluated based on findings such as drooling, saliva leakage, oral accumulation, and the need for frequent wiping, using a four-point scale (0 = none, 1 = mild, 2 = moderate, 3 = severe), with higher scores indicating greater impairment in saliva control. The scale was retrospectively derived from routine clinical documentation and had not undergone formal psychometric validation.

### 2.7. Disease Severity

Disease severity was assessed using the total score of the Amyotrophic Lateral Sclerosis Functional Rating Scale (ALSFRS). The ALSFRS consists of 12 items, each scored between 0 and 4, with a total score ranging from 0 to 48. Lower scores indicate more advanced disease severity. In this study, the ALSFRS total score was used as a variable representing disease severity in the analyses.

### 2.8. Statistical Analysis

Statistical analyses were performed using IBM SPSS Statistics version 30.0. Continuous variables were expressed as mean ± standard deviation or median (minimum–maximum), while categorical variables were presented as frequencies and percentages. Normality of continuous variables was assessed using the Shapiro–Wilk test.

Group comparisons were performed using the independent samples *t*-test, Mann–Whitney U test, or chi-square test, as appropriate. Associations between clinical dysphagia assessment tools and instrumental swallowing findings were analysed using Spearman’s rank correlation coefficient (ρ).

Multivariable regression analyses were performed in two steps to examine the independent associations between clinical dysphagia assessment tools and VFSS findings in patients with ALS. In Model 1, demographic and disease-related variables (age, sex, disease duration, onset type, and ALSFRS total score) were entered into the analyses. In Model 2, clinical dysphagia assessment tools (EAT-10, GUSS, RSST, and sialorrhea severity) were additionally included. PAS–Liquid and PAS–Semi-solid scores were analysed separately as dependent variables. Because PAS scores are ordinal in nature, additional ordinal logistic regression analyses (proportional odds model) were performed for PAS–Liquid and PAS–Semi-solid outcomes to provide a more appropriate modelling approach for ordinal dependent variables. Odds ratios (ORs), 95% confidence intervals (CIs), and *p*-values were reported for ordinal logistic regression models. Exploratory linear regression analyses were additionally retained to evaluate the consistency of associations across modelling approaches, and these findings were interpreted cautiously. Variance inflation factor (VIF) values were calculated to assess multicollinearity among variables.

Receiver operating characteristic (ROC) curve analyses were performed to evaluate the discriminative ability of EAT-10, GUSS, RSST, and sialorrhea severity scores for aspiration risk in patients with ALS. Aspiration risk was defined as PAS–Liquid ≥6, consistent with the original Penetration–Aspiration Scale classification, in which scores of 6–8 correspond to aspiration events involving material passing below the level of the vocal folds [18]. The area under the curve (AUC), sensitivity, specificity, and optimal cut-off values were calculated. Statistical significance was set at *p* < 0.05.

## 3. Results

### 3.1. Participant Characteristics

A total of 60 patients diagnosed with ALS were included in the study. The mean age of the study population was 60.73 ± 7.13 years. Of the participants, 63.3% (*n =* 38) had spinal-onset ALS and 36.7% (*n =* 22) had bulbar-onset ALS. The demographic and clinical characteristics of the study population are summarized in Table 1.

No statistically significant differences were found between the spinal- and bulbar-onset groups in terms of age, sex, or ALSFRS total scores (all *p* > 0.05). However, disease duration differed significantly according to onset type (*p* = 0.003).

### 3.2. Inter-Rater Reliability Analysis

PAS–Liquid and PAS–Semi-solid scores were evaluated by two independent raters who were blinded to each other’s assessments. Inter-rater reliability was assessed using the intraclass correlation coefficient (ICC). The ICC values were 0.93 (95% CI: 0.88–0.96) for PAS–Liquid and 0.91 (95% CI: 0.85–0.95) for PAS–Semi-solid, indicating a high level of agreement between raters.

### 3.3. Clinical and Instrumental Swallowing Assessment Findings

Descriptive statistics of clinical dysphagia assessment tools and videofluoroscopic swallowing study (VFSS) findings are presented in Table 2. No statistically significant differences were observed between the spinal- and bulbar-onset groups in terms of EAT-10, GUSS, RSST, and sialorrhea severity scores (all *p* > 0.05).

In the instrumental swallowing assessment, the bulbar-onset group demonstrated higher PAS scores and lower FOIS scores. A significant difference was observed for PAS–Semi-solid scores (*p* = 0.022), while PAS–Liquid scores showed a borderline difference between the groups (*p* = 0.050).

### 3.4. Correlation Analyses

Spearman correlation analysis was performed to evaluate the relationships between clinical dysphagia assessment tools and instrumental swallowing findings, and the results are presented in Table 3. In the overall sample, EAT-10 scores showed a strong positive correlation with PAS scores and a strong negative correlation with FOIS scores (ρ = 0.728, *p* < 0.001 and ρ = −0.772, *p* < 0.001, respectively). In analyses based on onset type, EAT-10 scores in the bulbar-onset group demonstrated a strong positive correlation with PAS–Liquid and a strong negative correlation with FOIS (ρ = 0.753, *p* < 0.001 and ρ = −0.859, *p* < 0.001, respectively). In the spinal-onset group, EAT-10, GUSS, RSST, and sialorrhea scores were found to be significantly correlated with PAS–Liquid (ρ = 0.689, *p* < 0.001; ρ = −0.762, *p* < 0.001; ρ = −0.713, *p* < 0.001; and ρ = 0.737, *p* < 0.001, respectively).

### 3.5. Multivariable Regression and Ordinal Logistic Regression Analyses

The independent associations between clinical dysphagia measures and VFSS findings identified in the multivariable regression analyses are presented in Table 4. For PAS–Semi-solid scores, sialorrhea severity showed a significant positive association (β = 0.455, *p* = 0.004). In addition, ALS onset type was significantly associated with PAS–Semi-solid scores (β = −0.213, *p* = 0.042). In the analysis of PAS–Liquid scores, ALS onset type was significantly associated with penetration–aspiration severity (β = −0.402, *p* = 0.002). Disease duration demonstrated a marginal positive association with PAS–Liquid scores (β = 0.198, *p* = 0.062), while lower ALSFRS scores also showed a marginal association with worse PAS–Liquid scores (β = −0.314, *p* = 0.050). In the FOIS analysis, GUSS scores (β = 0.504, *p* < 0.001) and ALSFRS total scores (β = 0.796, *p* < 0.001) were positively associated with functional oral intake level, whereas EAT-10 scores were negatively associated with FOIS scores (β = −0.286, *p* = 0.017). Disease duration and ALS onset type showed marginal associations with FOIS scores (*p* = 0.051 and *p* = 0.050, respectively). VIF values ranged from 1.005 to 4.683, indicating no severe multicollinearity, although moderate collinearity was observed among some clinically overlapping variables.

Additional ordinal logistic regression analyses were performed to account for the ordinal nature of PAS scores, and the findings are presented in Table 5.

In the PAS–Liquid model, lower ALSFRS scores, lower GUSS scores, lower RSST values, and higher sialorrhea severity were significantly associated with higher penetration–aspiration severity. In the PAS–Semi-solid model, bulbar-onset ALS, lower GUSS scores, and higher sialorrhea severity were significantly associated with higher PAS severity.

Overall, the ordinal logistic regression findings were broadly consistent with the correlation analyses and exploratory linear regression models.

### 3.6. ROC Analysis for Aspiration Risk

The results of the ROC analyses evaluating the discriminative ability of clinical dysphagia assessment tools for aspiration risk based on PAS–Liquid scores in patients with ALS are presented in Table 6. Among the evaluated clinical dysphagia assessment tools, GUSS demonstrated the highest AUC value (AUC = 0.89; 95% CI: 0.80–0.96), followed closely by RSST (AUC = 0.88; 95% CI: 0.79–0.95) and EAT-10 (AUC = 0.82; 95% CI: 0.71–0.92). Sialorrhea severity showed moderate discriminative ability (AUC = 0.74; 95% CI: 0.61–0.86). The optimal cut-off values for identifying aspiration risk were ≤9 for GUSS, ≤3 for RSST, ≥18 for EAT-10, and ≥2 for sialorrhea.

## 4. Discussion

This study investigated the relationship between clinical dysphagia assessment tools and VFSS findings in patients with ALS. The results demonstrated that several clinical assessment tools were significantly associated with penetration–aspiration severity and may provide clinically relevant information regarding swallowing safety. Findings were interpreted according to onset type, their relationship with instrumental measures, and their ability to reflect penetration–aspiration severity. To our knowledge, limited studies have directly compared multiple bedside dysphagia assessment tools with instrumental VFSS findings in patients with ALS. In addition to correlation analyses, the present study evaluated the discriminative ability of bedside clinical assessment tools for aspiration risk using ROC analyses, thereby providing clinically relevant information regarding their potential utility in routine dysphagia screening.

In comparisons based on onset type, patients with bulbar-onset ALS demonstrated higher penetration–aspiration severity and lower levels of functional oral intake. In particular, higher PAS–Semi-solid scores and lower FOIS scores suggest that swallowing safety is more severely affected in this group. Consistent with the literature, dysphagia has been reported to occur earlier and to progress more rapidly in patients with bulbar-onset ALS compared to those with spinal-onset disease [17,24]. This may be attributed to early involvement of bulbar muscles, leading to impairments in oral motor control and bolus preparation [14,15,25]. In contrast, no significant differences were observed between groups in EAT-10, GUSS, RSST, or sialorrhea scores, suggesting that clinical dysphagia symptoms may not differ according to ALS onset type and may not always correspond with instrumental swallowing findings [15,26].

Significant correlations were observed between clinical assessment tools and instrumental swallowing findings, particularly with PAS and FOIS scores. The strong negative correlations between PAS and RSST and GUSS scores suggest that these tests reflect clinical aspects of swallowing safety. The stronger correlations in the bulbar-onset group may be related to early involvement of the tongue, soft palate, and pharyngeal muscles, leading to greater impairments in bolus control and airway protection [17,24,27]. In contrast, bulbar functions have been reported to be relatively preserved for a longer period in patients with spinal-onset ALS [10,11].

The significant correlation between EAT-10 and PAS scores suggests that this scale reflects patient-perceived dysphagia and is associated with swallowing impairment. Similarly, patients with aspiration have been reported to exhibit higher EAT-10 scores [28]. Exploratory regression analyses demonstrated an association between EAT-10 and penetration–aspiration severity; however, this relationship was weaker than that of some other clinical tools, likely due to its subjective nature and limited ability to capture physiological aspects of swallowing safety. The stronger association between EAT-10 and PAS–Semi-solid in bulbar-onset ALS may be related to early bulbar muscle involvement, affecting bolus preparation, transfer, and pharyngeal clearance. However, as dysphagia in ALS may present with silent aspiration, subjective measures may not fully reflect aspiration severity [17,26].

GUSS and RSST scores showed significant associations with swallowing safety, with negative correlations observed between PAS–Liquid and both measures. Consistent with the literature, these tools appear to be clinically useful for assessing aspiration risk. In addition, ROC analyses demonstrated that GUSS and RSST showed good discriminative ability for aspiration risk in patients with ALS, suggesting that these bedside tools may help identify patients requiring further instrumental swallowing evaluation. Among the evaluated clinical dysphagia assessment tools, GUSS demonstrated the highest AUC value, followed closely by RSST. Exploratory regression analyses also suggested that GUSS had a relatively stronger association with PAS–Liquid scores compared with some other clinical measures, possibly due to its structured assessment across different consistencies and its sensitivity to airway protection, particularly for liquids. Similarly, RSST, which evaluates swallowing reflex and swallowing frequency, may reflect impairments in pharyngeal phase coordination [10,29]. Although the ROC findings support the clinical utility of GUSS and RSST, these tools should not be considered substitutes for VFSS. Rather, they may be useful as bedside screening tools to identify patients who require further instrumental evaluation. The moderate discriminative ability of sialorrhea severity also suggests that saliva control problems may reflect swallowing impairment in ALS, but sialorrhea alone is insufficient for determining aspiration risk. Furthermore, additional ordinal logistic regression analyses demonstrated findings broadly consistent with the exploratory analyses.

Another important finding was the observed association between lower ALSFRS scores and worse swallowing safety. The negative association between ALSFRS and PAS scores indicates that declining functional status is associated with worsening swallowing safety, consistent with previous reports of progressive dysphagia in ALS [24,30].

In our study, sialorrhea severity and FOIS scores were also found to be associated with dysphagia-related parameters. In ALS, sialorrhea is primarily related to reduced swallowing frequency and impaired oral motor control rather than excessive saliva production [15,16]. Weakness of the tongue and oral muscles may lead to saliva accumulation and impaired clearance, increasing the risk of aspiration. This suggests that bulbar muscle involvement may negatively affect saliva control, although evidence on differences across ALS onset types remains limited [24].

The significant associations between FOIS scores, which assess functional oral intake, and clinical dysphagia assessment tools indicate that the level of functional oral intake may reflect both patient-reported dysphagia symptoms and clinical swallowing performance. Videofluoroscopic studies have shown that pharyngeal residue and impaired laryngeal closure in patients with ALS may affect the level of oral intake [17,24].

FOIS should not be interpreted as a direct physiological measure of swallowing function. Unlike PAS, which reflects airway invasion during VFSS, FOIS represents a functional oral intake endpoint related to diet level, feeding dependence, and nutritional intake. Therefore, associations involving FOIS should be interpreted within the context of functional oral intake rather than instrumental swallowing physiology.

When the pathophysiology of dysphagia in ALS is considered, it is known that multiple neuromuscular changes affecting swallowing function occur. Videofluoroscopic studies have shown that patients with ALS may exhibit delayed laryngeal vestibule closure, reduced pharyngeal constriction, and limited upper esophageal sphincter opening [17]. Additionally, impairments in respiration–swallowing coordination have also been reported [16]. Therefore, the combined interpretation of clinical swallowing assessments and instrumental evaluations is important for planning dysphagia management in patients with ALS.

From a clinical perspective, the findings of this study suggest that bedside dysphagia assessment tools such as GUSS and RSST may be useful for the early identification of aspiration risk in patients with ALS, particularly in settings where instrumental swallowing assessment is not readily available. These tools may assist clinicians in identifying patients who require further instrumental swallowing evaluation and closer dysphagia management. However, bedside clinical assessments should not be considered substitutes for instrumental swallowing evaluation, and VFSS remains essential for the comprehensive assessment of swallowing safety whenever feasible.

Several limitations should be considered when interpreting the findings of this study. First, the relatively small sample size may have limited statistical power, particularly for subgroup comparisons. Second, due to the retrospective cross-sectional design of the study, causal relationships cannot be inferred. In addition, clinical dysphagia assessment tools may not fully capture all physiological aspects of swallowing function. Although VFSS is considered the gold standard for swallowing assessment, the use of the highest PAS score for each consistency may have overestimated penetration–aspiration severity compared with average swallow performance. However, this approach was preferred to maximize sensitivity for clinically relevant airway invasion. Another limitation is that the sialorrhea severity scale used in this study was clinician-derived and had not undergone formal psychometric validation, which may limit reproducibility and generalizability. Furthermore, although additional ordinal logistic regression analyses were performed to account for the ordinal nature of PAS scores, findings should still be interpreted cautiously due to the relatively small sample size. Finally, the limited number of studies directly comparing clinical dysphagia assessment tools with instrumental swallowing findings in ALS complicates interpretation of the present results. ROC findings should also be interpreted cautiously due to the relatively small sample size. Future studies with larger samples and prospective designs are needed to better clarify the clinical utility of bedside dysphagia assessment tools for identifying aspiration risk in patients with ALS. In addition, because bedside clinical assessments and VFSS examinations were performed on the same day, temporal proximity between assessments may have introduced incorporation bias and increased the likelihood of correlation inflation between clinical and instrumental swallowing measures.

## 5. Conclusions

The findings of this study suggest that several clinical dysphagia assessment tools are associated with instrumental swallowing safety findings in patients with ALS. Although instrumental swallowing assessments remain the gold standard for evaluating penetration–aspiration severity, their use may be limited in some clinical settings due to radiation exposure, poor patient cooperation, limited equipment availability, and cost-related factors. Therefore, bedside clinical dysphagia assessment tools may provide a practical and clinically useful approach for the early identification of aspiration risk.

In the present study, GUSS and RSST demonstrated strong associations with swallowing safety and good discriminative ability for aspiration risk in ROC analyses. EAT-10 also showed clinically relevant discriminative ability. These findings suggest the potential utility of bedside tools for identifying patients who may be at increased risk of aspiration. Nevertheless, clinical assessment findings should be interpreted together with instrumental swallowing assessments whenever feasible to ensure a comprehensive evaluation of swallowing safety.

## Figures and Tables

**Table 1:** Demographic and Clinical Characteristics of the Participants.

	Total (*n* = 60)	Spinal (*n* = 38)	Bulbar (*n* = 22)	*p*
Age (years)	60.73 ± 7.13	60.39 ± 7.21	61.05 ±7.14	0.737
**Gender, % (n)**				
Female	41.7 (25)	39.5 (15)	45.5 (10)	0.651
Male	58.3 (35)	60.5 (23)	54.5 (12)	
Disease duration (years)	2.92 ± 1.40	3.29 ± 1.03	2.27 ± 1.46	0.003 *
ALSFRS total score	23.07 ± 11.25	23.84 ±10.56	21.73 ±12.50	0.488

Comparisons between spinal- and bulbar-onset ALS groups were performed using the independent samples *t*-test for normally distributed continuous variables and the Mann–Whitney U test for non-normally distributed variables. Categorical variables were compared using the chi-square test. Data are presented as mean ± standard deviation or percentage (n). ALSFRS: Amyotrophic Lateral Sclerosis Functional Rating Scale. * *p* < 0.05 was considered statistically significant.

**Table 2:** Clinical Dysphagia Assessment Results and Instrumental Swallowing Findings.

Variable	Total (*n* = 60)				Spinal (*n* = 38)				Bulbar (*n* = 22)				*p*
	Min	Max	$\bar{X}$	SD	Min	Max	$\bar{X}$	SD	Min	Max	$\bar{X}$	SD	
EAT-10	1	38	18.80	9.26	1	32	17.29	8.48	5	38	21.41	10.13	0.108
GUSS	2	20	8.61	6.22	2	20	9.29	6.28	2	20	7.45	6.08	0.120
RSST	0	10	3.86	2.48	1	10	4.13	2.46	0	9	3.41	2.50	0.179
Sialorrhea	0	3	1.93	0.97	0	3	2.00	0.95	0	3	1.82	1.00	0.475
PAS—Liquid	1	8	5.33	2.52	1	8	4.92	2.54	1	8	6.05	2.36	0.050
PAS—Semi-solid	1	8	3.00	2.12	1	7	2.47	1.70	1	8	3.91	2.48	0.022 *
FOIS	2	7	3.96	1.97	2	7	4.32	1.94	2	7	3.36	1.89	0.044 *

Comparisons between spinal- and bulbar-onset ALS groups were performed using the independent samples *t*-test for normally distributed continuous variables and the Mann–Whitney U test for non-normally distributed variables. Categorical variables were compared using the chi-square test. Data are presented as minimum (Min), maximum (Max), mean ($\bar{X}$), and standard deviation (SD). EAT-10: Eating Assessment Tool-10; GUSS: Gugging Swallowing Screen; RSST: Repetitive Saliva Swallowing Test; PAS: Penetration–Aspiration Scale; FOIS: Functional Oral Intake Scale. * *p* < 0.05 was considered statistically significant.

**Table 3:** Correlations Between Clinical Assessment Tools and VFSS Findings.

Outcome	Group		EAT-10	GUSS	RSST	Sialorrhea
**PAS—Liquid**	Total	ρ	0.728	−0.717	−0.772	0.665
		n	60	60	60	60
	Bulbar	ρ	0.753	−0.750	−0.843	0.691
		n	22	22	22	22
	Spinal	ρ	0.689	−0.762	−0.713	0.737
		n	38	38	38	38
**PAS—Semi-solid**	Total	ρ	0.652	−0.687	−0.770	0.664
		n	60	60	60	60
	Bulbar	ρ	0.848	−0.795	−0.824	0.820
		n	22	22	22	22
	Spinal	ρ	0.495	−0.604	−0.741	0.697
		n	38	38	38	38
**FOIS**	Total	ρ	−0.772	0.630	0.662	−0.596
		n	60	60	60	60
	Bulbar	ρ	−0.859	0.602	0.632	−0.662
		n	22	22	22	22
	Spinal	ρ	−0.680	0.621	0.654	−0.640
		n	38	38	38	38

Correlations were calculated using Spearman’s rank correlation coefficient (ρ). All reported correlations were statistically significant (*p* < 0.001). EAT-10: Eating Assessment Tool-10; GUSS: Gugging Swallowing Screen; RSST: Repetitive Saliva Swallowing Test; PAS: Penetration–Aspiration Scale; FOIS: Functional Oral Intake Scale.

**Table 4:** Multivariable Linear Regression Analyses of Clinical Dysphagia Measures and VFSS Findings in Patients with ALS.

PAS–Semi-solid							
**Model 1. Demographic and Disease-Related Variables**							
**Variable**	**B**	**SE**	**β**	**t**	***p***	**95% CI**	**VIF**
Gender	−0.533	0.496	−0.125	−1.074	0.287	−1.526 to 0.461	1.010
Age	0.005	0.034	0.016	0.137	0.891	−0.064 to 0.074	1.005
Disease duration	0.627	0.187	0.415	3.352	0.001 *	0.252 to 1.003	1.151
ALS onset type	−2.038	0.539	−0.467	−3.779	<0.001 *	−3.119 to −0.958	1.142
**Model 2. Clinical Dysphagia Assessment Tools Added**							
**Variable**	**B**	**SE**	**β**	**t**	***p***	**95% CI**	**VIF**
Gender	−0.535	0.368	−0.125	−1.454	0.152	−1.273 to 0.203	1.035
Age	−0.050	0.027	−0.170	−1.873	0.067	−0.104 to 0.004	1.143
Disease duration	−0.294	0.190	−0.195	−1.545	0.128	−0.676 to 0.088	2.216
ALS onset type	−0.931	0.445	−0.213	−2.090	0.042 *	−1.825 to −0.037	1.450
EAT-10	0.052	0.034	0.225	1.533	0.131	−0.016 to 0.119	3.017
GUSS	−0.059	0.049	−0.173	−1.212	0.231	−0.157 to 0.039	2.836
RSST	−0.092	0.157	−0.107	−0.585	0.561	−0.406 to 0.223	4.683
Sialorrhea	0.994	0.327	0.455	3.042	0.004 *	0.338 to 1.650	3.122
ALSFRS	0.002	0.009	0.057	0.212	0.833	−0.016 to 0.020	4.683
PAS–Liquid							
**Model 1. Demographic and Disease-Related Variables**							
**Variable**	**B**	**SE**	**β**	**t**	***p***	**95% CI**	**VIF**
Gender	0.021	0.576	0.004	0.036	0.971	−1.133 to 1.175	1.010
Age	0.020	0.040	0.058	0.511	0.612	−0.060 to 0.101	1.005
Disease duration	0.950	0.217	0.531	4.372	<0.001 *	0.515 to 1.386	1.151
ALS onset type	−2.079	0.626	−0.402	−3.319	0.002 *	−3.334 to −0.824	1.142
**Model 2. Clinical Dysphagia Assessment Tools Added**							
**Variable**	**B**	**SE**	**β**	**t**	***p***	**95% CI**	**VIF**
Gender	−0.077	0.326	−0.015	−0.236	0.814	−0.732 to 0.578	1.035
Age	−0.028	0.024	−0.080	−1.174	0.246	−0.076 to 0.020	1.143
Disease duration	0.062	0.033	0.198	1.874	0.062	−0.004 to 0.128	2.798
ALS onset type	−1.402	0.626	−0.402	−3.319	0.002 *	−2.654 to −0.150	1.142
EAT−10	0.041	0.031	0.198	1.322	0.192	−0.021 to 0.103	3.017
GUSS	−0.071	0.045	−0.241	−1.578	0.121	−0.162 to 0.019	2.836
RSST	−0.186	0.144	−0.236	−1.292	0.202	−0.476 to 0.103	4.683
Sialorrhea	0.482	0.299	0.231	1.611	0.113	−0.119 to 1.083	3.122
ALSFRS	−0.013	0.007	−0.314	−2.008	0.050	−0.027 to 0.000	4.683
FOIS							
**Model 1. Demographic and Disease-Related Variables**							
**Variable**	**B**	**SE**	**β**	**t**	***p***	**95% CI**	**VIF**
Gender	0.083	0.406	0.021	0.205	0.839	−0.730 to 0.896	1.010
Age	−0.050	0.028	−0.182	−1.781	0.081	−0.107 to 0.006	1.005
Disease duration	−0.884	0.153	−0.632	−5.770	<0.001 *	−1.191 to −0.577	1.151
ALS onset type	1.814	0.442	0.448	4.107	<0.001 *	0.929 to 2.698	1.142
**Model 2. Clinical Dysphagia Assessment Tools Added**							
**Variable**	**B**	**SE**	**β**	**t**	***p***	**95% CI**	**VIF**
Gender	0.087	0.268	0.022	0.323	0.748	−0.451 to 0.624	1.035
Age	−0.020	0.020	−0.074	−1.039	0.304	−0.060 to 0.019	1.143
Disease duration	−0.277	0.139	−0.198	−2.000	0.051	−0.555 to 0.001	2.216
ALS onset type	0.652	0.324	0.161	2.012	0.050	0.001 to 1.304	1.450
EAT-10	−0.061	0.025	−0.286	−2.470	0.017 *	−0.110 to −0.011	3.017
GUSS	0.159	0.035	0.504	4.499	<0.001 *	0.088 to 0.230	2.836
RSST	0.020	0.114	0.025	0.173	0.863	−0.210 to 0.249	4.683
Sialorrhea	0.037	0.238	0.018	0.156	0.876	−0.440 to 0.515	3.122
ALSFRS	0.139	0.020	0.796	6.973	<0.001 *	0.099 to 0.179	4.683

Multivariable regression analyses were performed in two steps. Model 1 included demographic and disease-related variables, whereas Model 2 additionally included clinical dysphagia assessment tools. Exploratory linear regression findings were retained as supplementary analyses and interpreted cautiously. VIF values below 5 were considered acceptable. * *p* < 0.05 was considered statistically significant.

**Table 5:** Ordinal Logistic Regression Analyses of PAS Severity in Patients With ALS.

PAS–Liquid (Ordinal Logistic Regression)			
Variable	OR	95% CI	*p*
Gender	0.94	0.29–3.01	0.918
Age	0.98	0.91–1.05	0.531
Disease duration	1.21	0.86–1.71	0.274
ALS onset type	2.82	0.71–11.12	0.139
EAT-10	1.06	0.95–1.18	0.335
GUSS	0.83	0.68–1.00	0.049 *
RSST	0.55	0.33–0.94	0.028 *
Sialorrhea	2.96	1.13–7.77	0.027 *
ALSFRS	0.88	0.78–0.99	0.032 *
**PAS–Semi-Solid (Ordinal Logistic Regression)**			
**Variable**	**OR**	**95% CI**	***p***
Gender	0.81	0.25–2.65	0.734
Age	0.96	0.90–1.03	0.274
Disease duration	1.14	0.82–1.59	0.431
ALS onset type	8.84	2.02–38.66	0.004 *
EAT-10	1.03	0.92–1.14	0.633
GUSS	0.84	0.71–0.99	0.042 *
RSST	0.59	0.32–1.10	0.097
Sialorrhea	3.30	1.09–9.98	0.035 *
ALSFRS	0.92	0.82–1.02	0.124

Ordinal logistic regression analyses (proportional odds model) were performed to evaluate independent associations between clinical dysphagia assessment tools and PAS severity. Odds ratios (ORs) greater than 1 indicate increased odds of higher PAS severity. PAS: Penetration–Aspiration Scale; EAT-10: Eating Assessment Tool-10; GUSS: Gugging Swallowing Screen; RSST: Repetitive Saliva Swallowing Test; ALSFRS: Amyotrophic Lateral Sclerosis Functional Rating Scale. * *p* < 0.05 was considered statistically significant.

**Table 6:** ROC Analysis of Clinical Dysphagia Assessment Tools for Aspiration Risk in Patients With ALS.

Test	AUC	95% CI	Cut-off	Sensitivity (%)	Specificity (%)
EAT-10	0.82	0.71–0.92	≥18	78.4	76.2
GUSS	0.89	0.80–0.96	≤9	89.7	83.3
RSST	0.88	0.79–0.95	≤3	82.1	85.7
Sialorrhea	0.74	0.61–0.86	≥2	71.8	69.0

ROC analyses were performed to evaluate the discriminative ability of clinical dysphagia assessment tools for aspiration risk in patients with ALS. Aspiration risk was defined as PAS–Liquid ≥6, consistent with the Penetration–Aspiration Scale classification in which scores 6–8 represent aspiration below the vocal folds. AUC: Area Under the Curve; CI: Confidence Interval; PAS: Penetration–Aspiration Scale; VFSS: Videofluoroscopic Swallowing Study; EAT-10: Eating Assessment Tool-10; GUSS: Gugging Swallowing Screen; RSST: Repetitive Saliva Swallowing Test.

## Data Availability

The data supporting the findings of this study are available from the corresponding author upon reasonable request.
